# Influence of the Large-Span Pile-Beam-Arch Construction Method on the Surface Deformation of a Metro Station in the Silty Clay–Pebble Composite Stratum

**DOI:** 10.3390/ma16072934

**Published:** 2023-04-06

**Authors:** Tao Li, Yue Li, Tengyu Yang, Rui Hou, Yuan Gao, Bo Liu, Guogang Qiao

**Affiliations:** 1 School of Mechanics and Civil Engineering, China University of Mining and Technology, Beijing 100083, China; 2 Stage Key Laboratory for Geomechanics and Deep Underground Engineering, China University of Mining and Technology, Beijing 100083, China; 3 Beijing Municipal Construction Group Co., Ltd., Beijing 100032, China

**Keywords:** large-span pile-beam-arch, surface deformation, numerical simulation, pilot tunnel excavation scheme, arch secondary lining construction

## Abstract

The Pile-beam-arch (PBA) method is a new and effective construction method for the urban metro station. It is the key to ensuring the safe construction of the station to clarify the influence of PBA method construction on surface deformation under unfavorable geological and large span conditions. Based on a station of Beijing subway, this paper studies the surface deformation law of the large-span PBA method in different construction stages under silty clay–pebble composite stratum by means of FLAC 3D numerical analysis and field monitoring of level. Then the influence of the excavation scheme of the pilot tunnel and the construction scheme of the secondary lining of the arch on the surface deformation is simulated and analyzed. The results show that, through numerical simulation, the ratio of pilot tunnel excavation: pile-beam construction: vault initial support construction: vault secondary lining construction is about 5:1.1:3.3:0.6. The settlement deformation mainly occurs in the excavation stage of the pilot tunnel. Through the comparative analysis of the field monitoring results and the numerical simulation results, it can be seen that the two results are highly consistent, which verifies the accuracy of the numerical simulation results. The pilot tunnel excavation scheme of excavating the middle first and then excavating both sides, first through the upper layer and then through the lower layer, and the scheme of one-time construction of the secondary lining of the arch are better. The research results promote the further maturity and perfection of large-span PBA method construction under unfavorable geology and provide reference for similar projects.

## 1. Introduction

With the increasingly serious problem of urban traffic congestion, the metro as an effective solution is being widely built in the world’s major cities [1,2]. The metro station is a key part of metro construction. Most of them are built in dense urban areas with complex geological conditions. Excessive stratum settlement is easily caused in the construction process, which adversely affects the safety performance of surrounding buildings [3,4,5,6,7,8,9,10,11]. Improper construction can even lead to serious accidents such as ground collapse and the collapse of surrounding buildings. The safe construction of metro stations has always been a major concern.

At present, the construction methods of metro stations at home and abroad mainly include the open-cut method, cover excavation method, shield method and shallow cover excavation method. Compared with other construction methods, the shallow buried concealed excavation method has become the mainstream choice for the construction of metro stations due to its flexible structure and small impact on the city and environment. The shallow buried excavation method can be divided into full section method, CD method, CRD method, side cavity method, middle cavity method, PBA method and so on, according to different construction methods [12]. Among them, the PBA method combines the advantages of the cover excavation method and subsurface excavation method, which can greatly control the stratum deformation during the construction of the metro station while ensuring construction efficiency [13,14,15]. The PBA method was first applied in the construction of Dongdan Station of Beijing Metro in 1992 and achieved excellent application results [16]. At present, it has been widely used in metro station construction in China [17,18,19,20].

Many scholars and engineers have done extensive research on the PBA method. Through numerical simulation [16,19,20,21,22,23], field monitoring [7,24,25,26] and model test [20,27], the construction, support parameters, deformation and mechanical response of PBA metro station are studied comprehensively. However, with the continuous development of metro station construction in the direction of large span and large section, new requirements and challenges have been brought to the PBA method, and the construction difficulty of the station will be further aggravated when crossing complex strata. At present, the research on large-span and large-section PBA station under complex stratum conditions is not sufficient. The influence of different construction stages of the large-span and large-section PBA station on surface settlement under complex stratum conditions and the optimal construction steps in key construction stages are not thoroughly studied.

**Figure 1:**
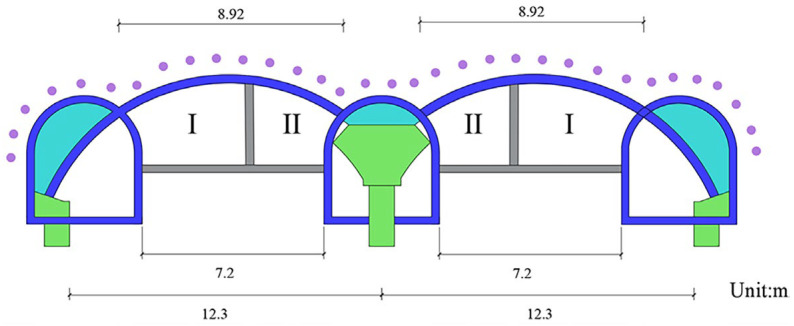
Schematic of the large-span section of Qinghuadongluxikou Station.

This paper takes the Qinghuadongluxikou Station project of Beijing Metro Line 15 as the research object. As shown in Figure 1, the single span of the supporting arch reaches 12.3 m, which is nearly twice the span of the supporting arch under conventional conditions, forming a typical case of large-span PBA. In addition, the station is built in silty clay–pebble composite strata. The engineering properties of the upper silty clay stratum and the lower pebble stratum are very different. Among them, the upper silty clay has poor self-stability and is prone to collapse during construction. The lower pebble stratum has small cohesion and is prone to collapse [28]. The composite stratum is a typical bad geological condition, and the station under this stratum is easy to cause excessive subsidence of the surface during construction. Therefore, it is the key to ensure the safe construction of the station to clarify the influence of PBA method construction on surface deformation under bad geological and large span conditions. In this paper, the influence of each construction stage of the large-span PBA method on surface deformation under silty clay–pebble composite stratum is explored by combining numerical simulation with field measurement. Then the key construction stage, which is easy to cause surface deformation, is studied; that is, the influence of three different excavation factors on surface deformation in the construction stage of the pilot tunnel is simulated and analyzed, and the optimal excavation sequence of the pilot tunnel is obtained. Finally, according to the engineering characteristics of the large-span PBA method, the two construction schemes of the arch secondary lining are simulated and compared. The research results enrich the relevant knowledge of the influence of large-span PBA method construction on the surface deformation of subway stations under bad geology. The optimal construction scheme obtained in this paper provides a reference for the design and construction of subway stations similar to the PBA method.

## 2. Project Overview

### 2.1. Overview of Station Structure

**Figure 2:**
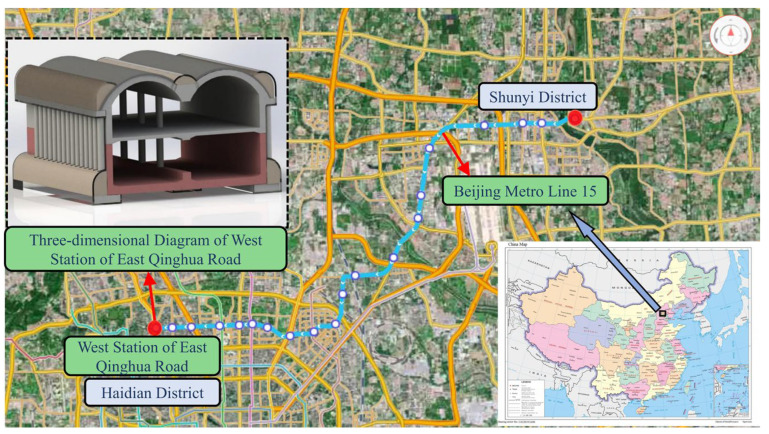
Schematic diagram of the location of Qinghuadongluxikou Station.

**Figure 3:**
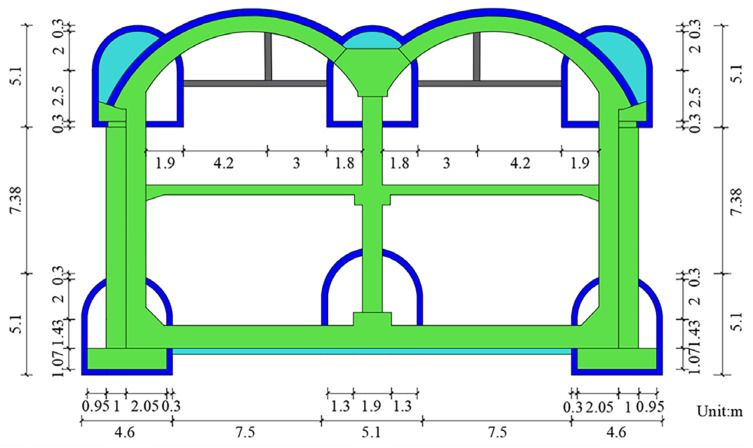
Cross-sectional dimensions of Qinghuadongluxikou Station.

Qinghuadongluxikou Station is the terminal station of Beijing Metro Line 15, which is located along Tsing Hua East Road in an east–west direction. The station measures a total of 236.4 m in length, 24.6 m in width for a standard double-arch section, 16.78 m in height, 9.5 m in thickness for the soil that covers the roof plate and about 26.0 m in depth for the bottom slab. Additionally, the main body of the station is a one-column, two-span, double-deck, lateral platform structure. The station hall is located on the first underground floor, while the platform is located on the second underground floor. A schematic showing the location of the station is presented in Figure 2, and the standard cross-sectional dimensions of the station are shown in Figure 3.

According to the preliminary survey results of the construction area, the stratum where the main structure of Qinghuadongluxikou Station is located (from the surface into the ground) can be divided into the (a) artificial soil layer, (b) Quaternary Holocene alluvial–pluvial deposit and (c) Quaternary Late Pleistocene alluvial–pluvial deposit based on the sedimentary strata ages and genetic types. In addition, the project can be divided into eight soil layers, and the specific parameters are shown in Table 1. The terrain and climate characteristics of the area where the subway station is located are shown in Table 2.

### 2.2. Station Construction Procedure

**Figure 4:**
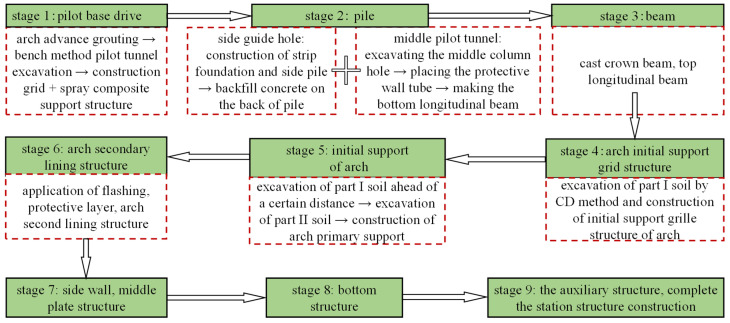
Construction procedure of PBA method.

**Figure 5:**
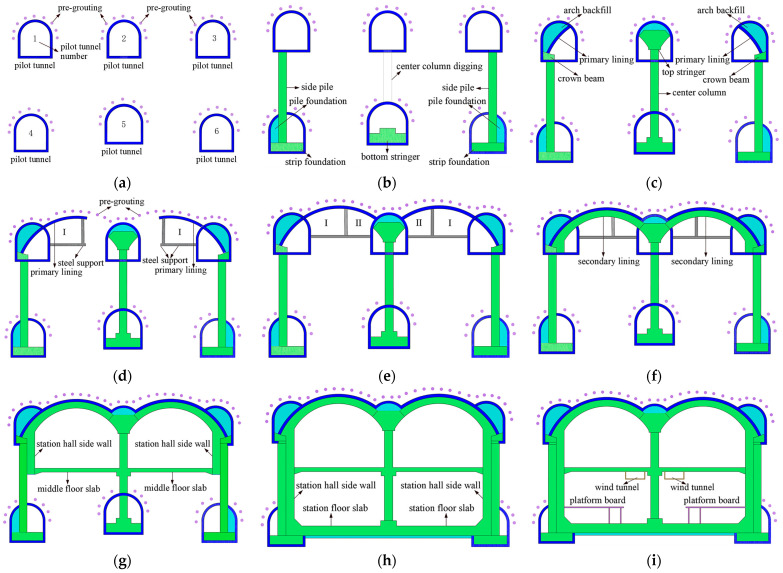
Schematic of the main construction process of Qinghuadongluxikou Station: (**a**) stage 1; (**b**) stage 2; (**c**) stage 3; (**d**) stage 4; (**e**) stage 5; (**f**) stage 6; (**g**) stage 7; (**h**) stage 8; (**i**) stage 9.

Qinghuadongluxikou Station is a two-story and two-span station, and the main construction procedure adopted to build it can be divided into nine stages. The detailed construction steps are explained below and can be observed in Figure 4. The Schematic of the main construction process of Qinghuadongluxikou Station is shown in Figure 5.

## 3. Construction of Numerical Model

In this study, FLAC 3D 6.0 numerical analysis software was used to simulate the subway station. To simulate all the engineering procedures more accurately and to improve the efficiency of the numerical model analysis as much as possible, this study makes some reasonable assumptions and simplifications of the actual engineering during the construction of the numerical analysis model:

1. The numerical model assumes that the soil is homogeneous and isotropic, and the same stratum in the soil is uniform, of equal thickness, and horizontal.
2. In this study, the load mainly includes the soil, structure weight and ground based on the addition of overload of 20 kPa.
3. In the pre-grouting consolidation simulation, the material parameters of the surrounding soil around the tunnel face were improved to simulate its effect equivalently. First, the pre-grouting part of the pilot tunnel was equivalent to the fan-shaped reinforcement with a thickness of 1 m combined with the actual construction situation. Second, the deep-hole grouting part of the arch is equivalent to the fan-shaped reinforcement of the 2-m thickness in consideration of the actual construction situation.
4. Grid mechanics simulation: As this is a steel, grid-shotcrete, composite supporting structure, its mechanical properties are relatively complex. Typically, it is usually simplified by the equivalent stiffness method in simulations,
  (1) $$E=E_{0}+\frac{S_{g}\times E_{g}}{S_{0}}$$
  where E is the elastic modulus of the entire structure; $E_{0}$ is the elastic modulus of the concrete; $E_{g}$ is the elastic modulus of steel; and $S_{0}$ is the area of the concrete per unit area.
5. Supporting structure simulations of the CD method: The joint steel is used for support, and the horizontal interval is the excavation spacing. The force is mainly based on the vertical load and transversal flexure, so it can serve as an equivalent wall based on the principle of equal flexural rigidity,
  (2) $$E_{s}I_{s}=E_{w}I_{w}=\frac{1}{12}bh^{3}$$
  where $E_{s}$ is the elastic modulus of steel; $I_{s}$ is the moment of inertia of steel; $E_{w}$ is the elastic modulus of the equivalent diaphragmatic wall; $I_{w}$ is the moment of inertia of the equivalent diaphragmatic wall; b is the I-beam erection span; and h is the thickness of the equivalent diaphragmatic wall.
6. In the numerical simulation of this study, the Mohr-Coulomb model was selected as the constitutive model of the soil, and each soil layer was considered as an ideal elastoplastic body. The lining, beam and plate all adopted the elastic model. In addition, the mechanical characteristics of the side piles were simulated by the pile element. The interior column was mainly subjected to vertical loads, and there was no coupling effect with the surrounding soil after construction. However, the two ends of the column have similar constraints to those of the beam element. Thus, the beam element is used to simulate the interior column.

**Figure 6:**
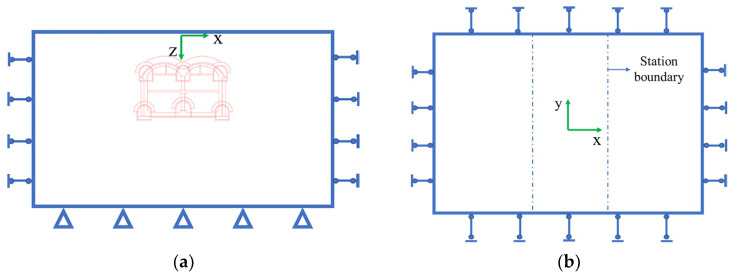
Schematic of the model boundary: (**a**) Front and (**b**) vertical views.

**Figure 7:**
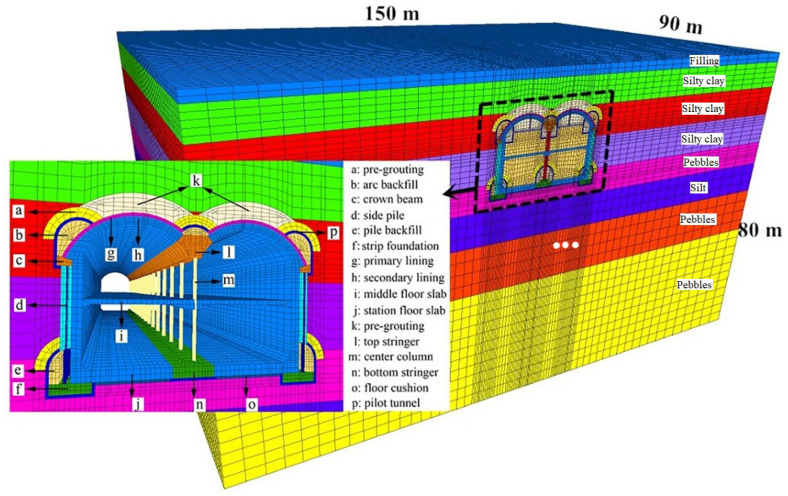
Structure and stratum numerical model of Qinghuadongluxikou Station.

**Figure 8:**
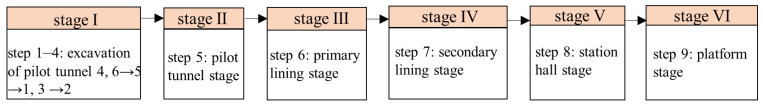
Simulated construction steps.

In this study, the main body of the station between shafts No. 1 and No. 2 was used, according to this numerical simulation section. The model selected a range that was equal to three times the excavation range as the model boundary, and the model size was 150 m × 90 m × 80 m. The model boundary conditions were all set as displacement boundary conditions, whereby the upper surface was a free boundary. Additionally, the bottom boundary of the model had a fixed displacement in the x, y and z directions; the displacement in the x direction of the right boundary was fixed, and the displacements in the y direction of the front and rear boundaries of the model were also fixed. Figure 6 and Figure 7 are schematics of the model boundaries and the model itself, respectively. The simulated construction steps are shown in Figure 8.

In the numerical simulation, the soil mechanical parameters were selected based on the parameters suggested by the reconnaissance report of this engineering structure. The parameters of the supporting structure are determined, according to the actual materials and design specifications. The detailed parameters of each supporting structure involved in the numerical simulation of the station are presented in Table 3.

**Figure 9:**
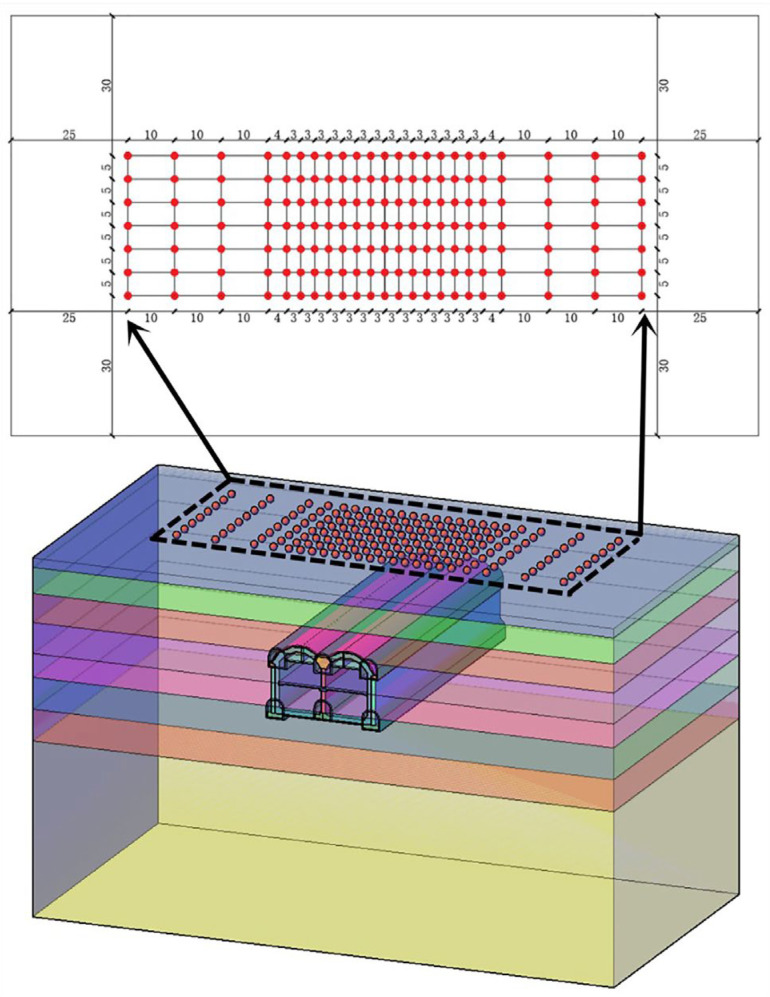
Schematic of surface monitoring site arrangement.

In the numerical simulation, to avoid the influence of the model boundary on the simulation results, the influencing scope of the excavation face at 45° was considered in conjunction with relevant soil mechanical principles, and the middle part of the model was generally selected as the monitoring range part. The surface monitoring site arrangement of the model is illustrated in Figure 9.

## 4. Results

### 4.1. Analysis of the Surface Displacement in the Entire Construction

#### 4.1.1. Vertical Displacement Analysis of Ground Surface

**Figure 10:**
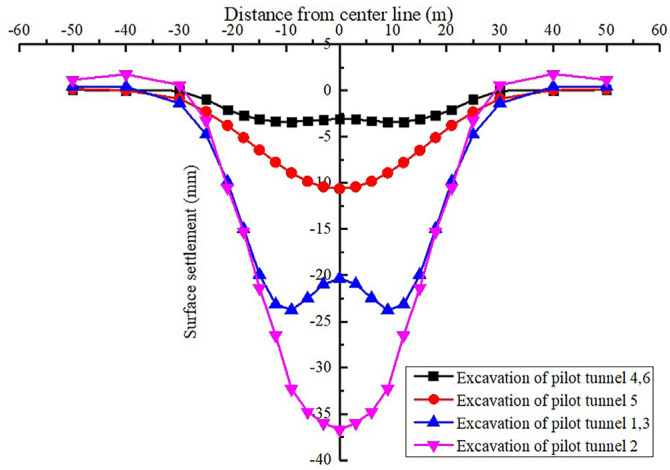
Plots of settlement troughs during pilot tunnel construction.

**Figure 11:**
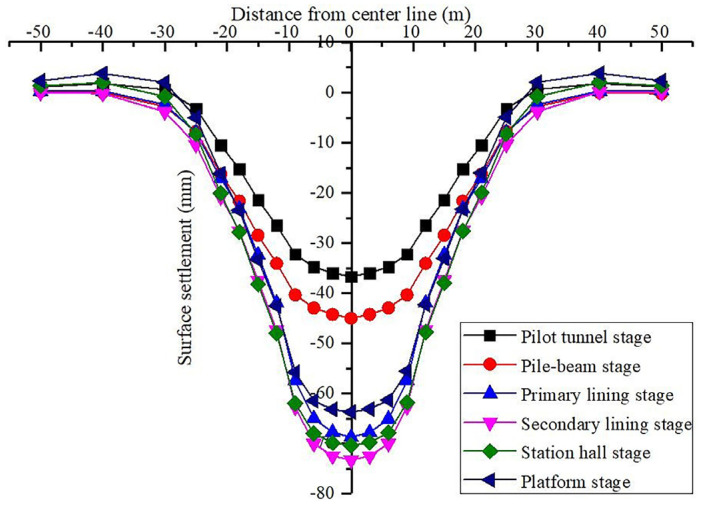
The curve of ground settlement in each stage of station Construction.

**Figure 12:**
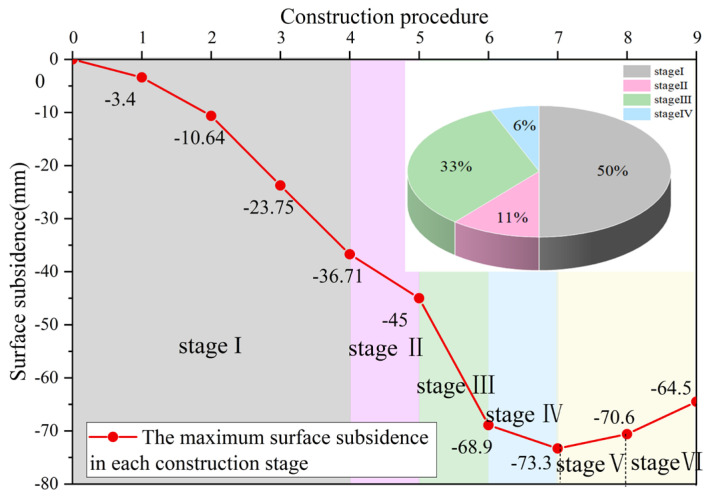
Relationship between surface subsidence and construction steps.

The surface subsidence variation curve during pilot tunnel excavation is shown in Figure 10. The surface settlement curve of each stage of the station construction is shown in Figure 11. The relationship between surface subsidence and construction steps is shown in Figure 12.

Through Figure 10 and Figure 12, we can see the surface subsidence law of the pilot tunnel excavation stage:

1. When the pilot tunnel is excavated for 4, 6→5→1, 3→2, the maximum ground settlement is 3.4 mm, 10.64 mm, 23.75 mm and 36.71 mm, respectively. It indicates that the excavation of the upper pilot tunnel has more obvious influence on the surface settlement than that of the lower pilot tunnel.
2. In the excavation stage of the pilot tunnel on both sides, the maximum point of the entire ground settlement is not on the medial axis of the station, which is a unique feature of large-span PBA construction. However, single-groove settlement usually occurs in the construction of the PBA with the conventional span. The main reason for this difference is that the pilot tunnel spacing of the same layer in the large-span PBA method is larger than that in other cases, and the multicavern effect between pilot tunnels is weaker than that for the small pilot tunnel with clear spacing.

From Figure 11 and Figure 12, we can see the law of surface subsidence in each stage of station construction:

1. During the entire building period of the station, the maximum value of surface settlement always appears at the middle line of the station structure. The settlement in the range of 30 m around the central line of the station has obvious changes with different construction stages. The surface deformation outside 30 m does not change significantly with different construction stages. The overall variation trend of the settlement trough curve is generally consistent with the Peck formula.
2. During the entire construction period, the ground settlement in the first four construction stages exhibits an increasing trend, and the range of the settlement groove exhibits a widening trend. In the last two construction stages, the ground settlement depicts a decreasing trend, and the range of the settlement groove also appears to be narrowing. This shows that in the large-span pile-beam-arch method’s construction process, the process prior to the arch secondary lining is the primary stage that contributes to surface deformation due to settlement, and the platform layer and station hall layer construction are the primary stages that contribute to surface uplift.
3. With the exception of the uplift stage, the ground settlement brought on by the building of the pilot tunnel makes up 50% of the total station settlement, while the ground settlement brought on by the construction of the pile-beam supporting system makes up 11% of the total. The ground settlement generated by the second lining of the arch construction accounts for 6% of the overall settlement, while the ground settlement induced by the initial arch supporting construction accounts for 33% of the total station settlement. The findings indicate that the settlement ratio of the excavation of the pilot tunnel: pile-beam construction: arch initial support: arch secondary lining is about 5:1.1:3.3:0.6. Settlement deformation mainly occurs in the pilot tunnel excavation stage. Therefore, the actual construction should focus on the prevention and control of the ground subsidence pilot tunnel excavation stage.

#### 4.1.2. Horizontal Displacement Analysis of Ground Surface

**Figure 13:**
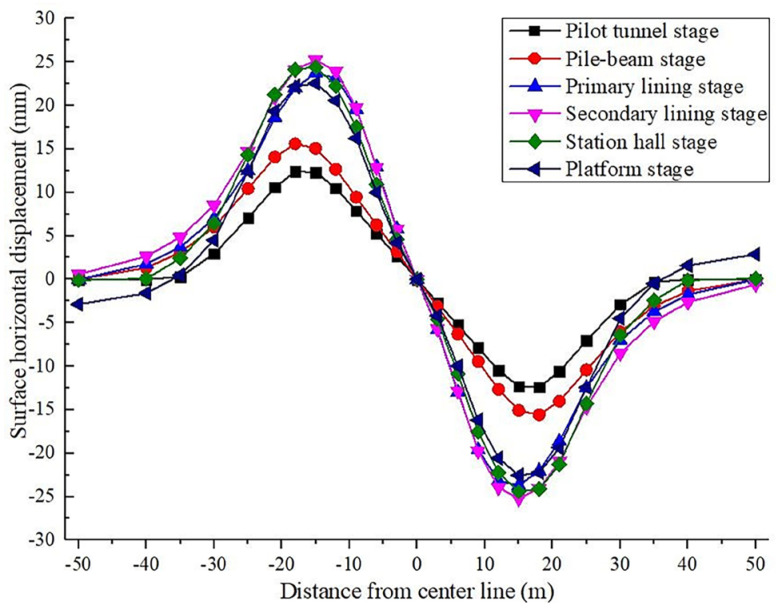
Horizontal displacement of station construction surface.

As shown in Figure 13, in each stage of the construction of the large-span PBA method, the horizontal displacement of the ground surface yields an antisymmetric figure with the ground center as the center of symmetry. In the first four phases, the horizontal displacement of the ground surface gradually increases as the construction progresses, and the peak value gradually draws closer to the midline of the station structure. The peak value of surface horizontal displacement in each stage is distributed between 15–18 m from the midline of the station structure. After the second lining of the arch was built, the horizontal displacement of the ground surface reached the maximum value of 25.2 mm. Additionally, the ground surface’s horizontal displacement gradually decreased in the last two stages. This is attributed to the fact that the structure exhibits an uplifting trend after the excavation of the station and the extrusion of the overlaying soil, which results in the rebounding of the ground surface horizontal displacement value.

### 4.2. Comparison of Field Measurements and Simulation Results

**Figure 14:**
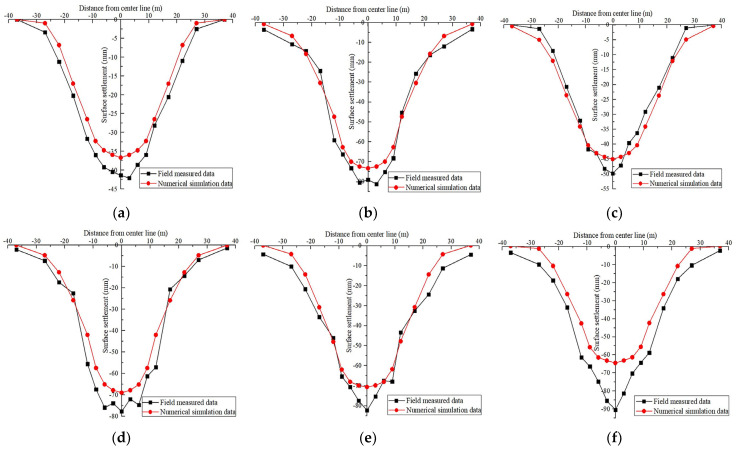
Comparison of simulated and measured ground subsidence at various construction stages: Completion of (**a**) pilot tunnel construction, (**b**) pile-beam construction, (**c**) first lining of the arch construction, (**d**) second lining of the arch construction, (**e**) station hall construction and (**f**) platform layer construction.

In the field monitoring project of the subway station, the surface subsidence is monitored for a long time by the level. As can be observed from the comparison in Figure 14, numerical simulation analysis results are relatively good in the first construction stages. In the last two construction stages, because of the unloading of soil pressure in the tunnel, the simulated value shows an upward trend, while the field measurement values still decrease slightly. The reasons for this phenomenon are the following: (a) the displacement caused by earth pressure unloading in the tunnel is difficult to transfer to the surface; (b) soil is not elastomer; and (c) in the numerical simulation, the time effect of soil excavation and support structure sealing loop cannot be considered.

**Figure 15:**
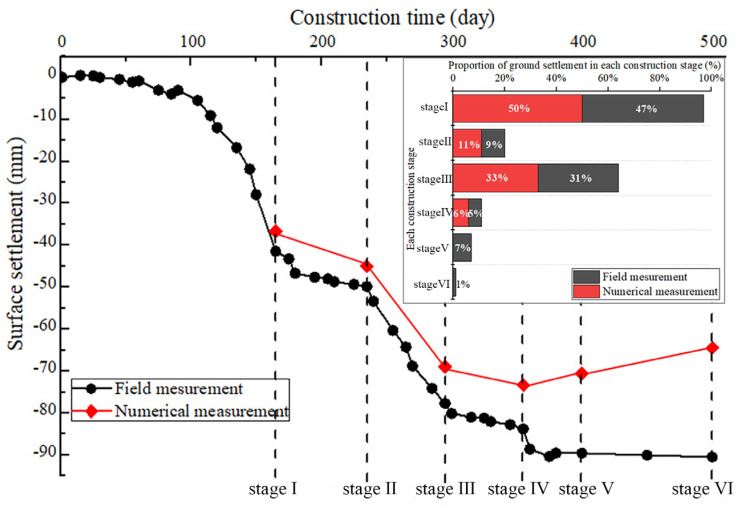
Variation of the surface settlement of Qinghuadongluxikou Station during the construction period.

Figure 15 shows the ground settlement variation results over the course of the station’s building and compares the numerical simulation results at the critical time nodes. The slope of the curve indicates that the pilot tunnel and arch constructions were significant stages of the surface deformation process brought on by the PBA method during construction, whereas the deformations during the other stages were quite smooth. In addition, with the exception of the station’s construction stage, the variation trend of ground settlement over the entire period was more in line with the calculated outcomes of the numerical model. The previous paragraph fully analyzed the reasons for the relevant differences.

### 4.3. Comparative Study on Different Excavation Schemes of Pilot Tunnels

The excavation of the pilot tunnel is the crucial construction phase that results in surface subsidence, according to the analysis of Section 4.2. Therefore, the following is a comparative analysis of the excavation schemes for the pilot tunnel using the principle of the control variable method. This paper systematically studies the effect of three main factors on the ground subsidence law when excavating the large-span PBA pilot tunnel, which is respectively through construction or synchronous stagger construction, horizontal excavation sequence and vertical excavation sequence. Finally, the optimal pilot tunnel excavation scheme, suitable for the large-span PBA method, is selected. The specific research plan is shown in Table 4.

**Figure 16:**
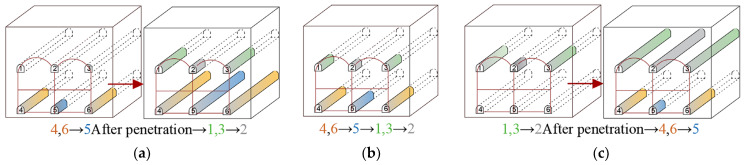

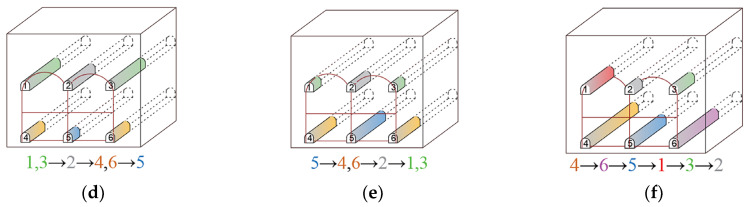
Diagram of six pilot tunnel excavation schemes: (**a**) plan 1; (**b**) plan 2; (**c**) plan 3; (**d**) plan 4; (**e**) plan 5; (**f**) plan 6.

**Figure 17:**
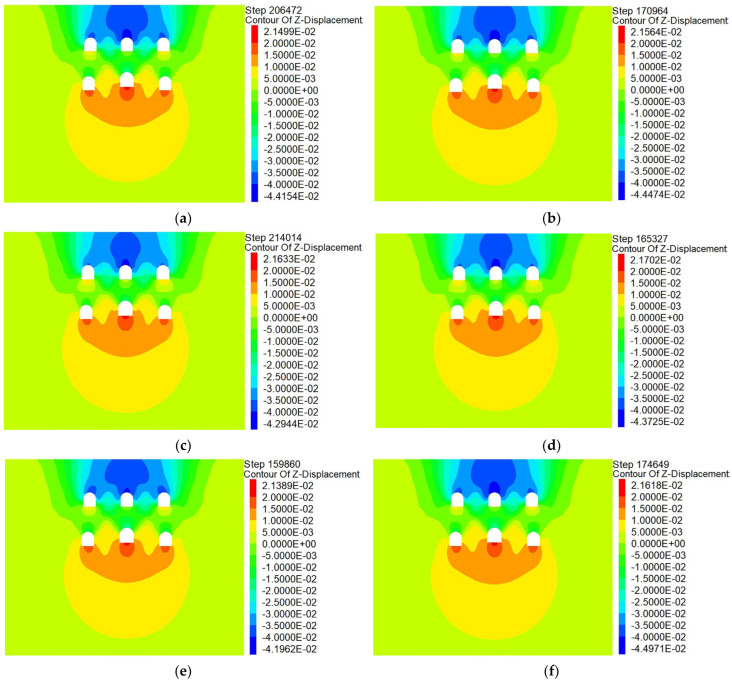
Vertical displacement clouds of different construction schemes: (**a**) plan 1; (**b**) plan 2; (**c**) plan 3; (**d**) plan 4; (**e**) plan 5; (**f**) plan 6.

**Figure 18:**
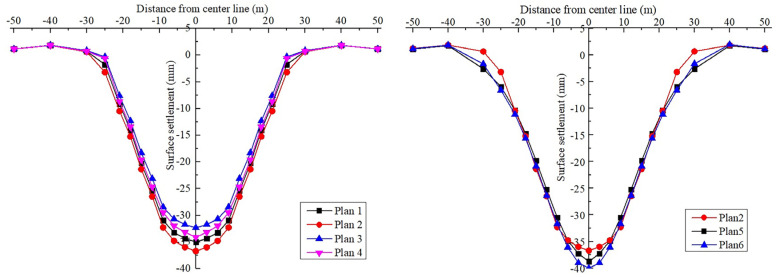
Comparison of surface settlements of different schemes.

Figure 16 displays the plan for excavating the six pilot tunnels. Figure 17 shows the vertical displacement cloud diagram for the six pilot tunnel excavation schemes. The deformation curve of the six plans in Figure 18 is drawn by extracting the model monitoring site data.

1. Whether the pilot tunnel of each layer is constructed separately.

It can be learned from the vertical displacement clouds of plans 1–4 that the settlement contour range of plan 1 and plan 2, which excavate the lower pilot tunnel first, is wider than that of plan 3 and plan 4, which excavate the upper pilot tunnel first. From the surface settlement curves of plans 1–4, it can be learned that the maximum surface settlement of plans 1–4 is 34.98 mm, 36.69 mm, 32.33 mm and 34.09 mm, respectively. Control univariate for comparative analysis, respectively, compared plan 1 and plan 2, plan 3 and plan 4. It can be concluded that during the construction process of the pilot tunnel, the surface settlement value caused by constructing another layer of the pilot tunnel after completing the construction of one layer of the pilot tunnel will be smaller than the surface settlement value caused by the simultaneous construction of a certain staggered distance between pilot tunnels. Comparing plan 1 with plan 3, it can be concluded that if the scheme of constructing another layer of the pilot tunnel after the completion of the first layer of the pilot tunnel is adopted. So, first through the upper guide hole will be smaller than the ground settlement value caused by first through the lower guide hole.

2. Horizontal excavation sequence

Control single variables for comparative analysis, comparison plan 2, plan 5 and plan 6. Plan 2 and plan 5 had approximately the same range of surface settlement, and plan 6 had a slightly wider range of surface settlement than the first two. It is also evident that the surface subsidence range of plan 5 is the widest; plan 6 is the second; and plan 2 is the narrowest. Through the surface subsidence curve of plans 2, 5 and 6, it can be known that the maximum surface subsidence of plans 2, 5 and 6 is 36.69 mm, 35.32 mm and 38.07 mm, respectively. It shows that plan 5 is better than plans 2 and 6. It can be concluded that, in the construction of the large-span PBA method, the same layer of the pilot tunnel is better to adopt the symmetrical excavation method of first middle and later sides.

3. Vertical excavation sequence

Control single variables for comparative analysis, comparison plan 2, plan 4. The surface settlement curves of plan 2 and plan 4 show that the maximum surface settlement of plan 2 and plan 4 are 36.69 mm and 34.09 mm, respectively, which indicates that plan 4 is better than plan 2. It can be concluded that, in the construction of the large-span PBA method, the plan of excavating the upper guide hole first is better than that of excavating the lower guide hole first.

For the results of the numerical simulation of six different plans, the theoretical maximum surface settlement value and settlement trough width are obtained by fitting, and the ground loss rate caused by each construction scheme is calculated. The results are shown in Table 5.

Through the above numerical simulation results analysis, surface subsidence curve fitting analysis and formation loss rate analysis, it can be clearly concluded that the following.

1. It is better to control the development of surface settlement after the construction of one layer of pilot tunnel first and then another layer of pilot tunnel than the staggered construction plan of two layers of pilot tunnels at the same time, and the plan of penetrating the upper layer of pilot tunnel first is better than the plan of penetrating the lower layer of pilot tunnel first in construction.
2. For the excavation sequence of the same layer pilot tunnel, the symmetrical excavation method of first middle and then side is better than the symmetrical excavation method of first side and then middle. The method of staggered excavation of single pilot tunnel has the worst effect on controlling ground settlement deformation and will cause uneven settlement on both sides of the station.

The above analysis results, first the middle and then the side, first through the construction of the upper layer and then the construction of the lower layer, is the best construction plan for the excavation of the pilot tunnel.

### 4.4. Comparative Study on Different Construction Schemes of the Second Arch Lining

**Figure 19:**
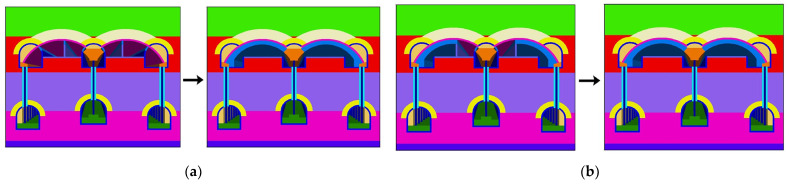
Schematic of different schemes for second lining simulation of arch: Second lining of the arch was completed in (**a**) one attempt and (**b**) two attempts to complete the program simulation.

After the initial arch support is completed, the side wall of the pilot tunnel is broken to construct the second lining of the arch. Given that the supporting arch span of this case is very large and reaches 12.3 m, there are two types of construction of the second lining of the arch. The first is the use of the inverted support method to construct the second lining of the arches in two times according to parts I and II divided by central partition wall in the initial arch support period. The second is the completion of the second lining of the arch in a single attempt after the removal of the central partition wall in the CD method. This part will simulate the above second lining of arch construction methods separately and compare the differences between the two construction methods. The simulations of the two methods are presented in Figure 19.

#### 4.4.1. Analysis of the Difference of the Influences on Surface Displacement

**Figure 20:**
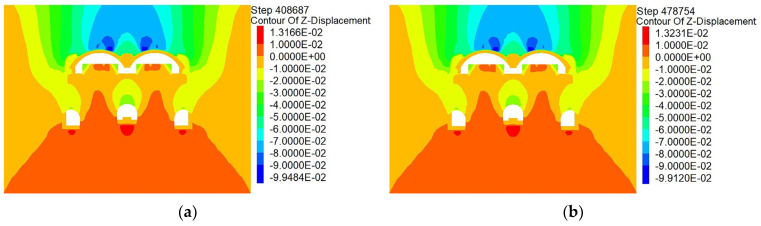
Vertical displacement cloud diagrams of different schemes. Arch completed in (**a**) one and (**b**) two attempts.

**Figure 21:**
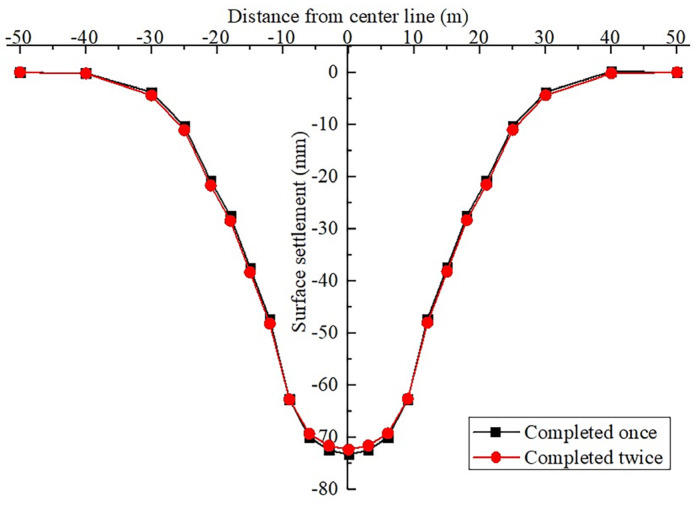
Surface subsidence curve after the completion of the two construction schemes.

According to the vertical displacement clouds shown in Figure 20, after completing the two types of the second lining of arch construction, the vertical displacement cloud diagrams are very similar after the completion of the two schemes. Furthermore, the ground settlement range does not change significantly. This shows that the initial supporting structure yields a good supporting effect, and the two schemes of the second lining of the arch have minor influences on the ground settlement. Following the extraction of the model monitoring site data of the two schemes, the relevant ground settlement curves are plotted in Figure 21. As shown, the ground settlement curves caused by the construction of the two schemes are very similar, and the differences are only reflected within distances of 9 m from the midline of the structure. Accordingly, the ground settlement value of the arch completed in one attempt is slightly greater than that of the arch completed in two attempts. Outside the 9 m range, the results are opposite. The reason for this difference is attributed to the fact that the supporting arch cross-section of the arch completed once is larger than that of the arch completed twice, so the maximum ground settlement value is slightly larger. However, based on comparisons, it can be concluded that the influences of the two different schemes on the changes of the ground settlement value are approximately the same.

#### 4.4.2. Analysis of Structural Stability Differences

**Figure 22:**
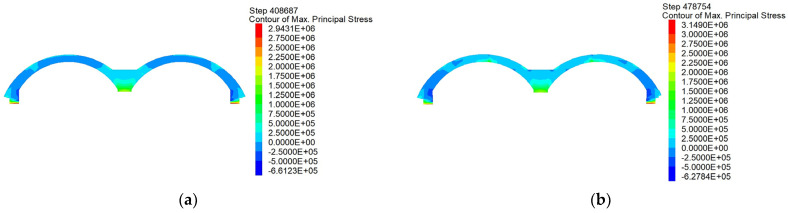
Comparison of maximum principal stresses of the arches completed in (**a**) one and (**b**) two attempts.

The maximum principal stress clouds (Figure 22) are completed by the second lining of the arch construction. It is obvious that, in the scheme of the arch completed once, the arch stress is evenly distributed; the arch is mainly in a compressed state; and the foot of both arch ends has the highest compressive stress value. In addition, the regions in which the positive and negative stresses occur are at the junction of the arch, top stringer and side pilot tunnel. However, the scheme of construction completed in two attempts presents an uneven distribution of stress. The primary disadvantage is that there is an obvious tension stress zone in the supporting part of the original central partition wall, which will have a very adverse effect on the stability of the concrete structure. In the process of the construction of the structure with the PBA method, the top plate adopts an arch structure to benefit maximally from the compression advantages so that the enforced load can be transmitted to both sides of the arch foot and so that the arch structure focuses on the compressive stress. Therefore, in terms of the force angle, the scheme of the construction completed in two attempts is significantly worse than the scheme of the arch completed in a single attempt.

**Figure 23:**
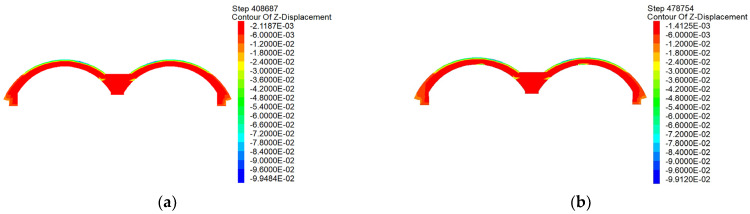
Comparison of the most vertical displacements of the arches completed in (**a**) one and (**b**) two attempts.

From the vertical displacement clouds of the arch shown in Figure 23, the scheme of the arch completed in a single attempt yields a uniform deformation. The average settlement is approximately 3 mm, which also confirms the uniformity of the aforementioned stress distribution, thereby indicating that the force and deformation in the scheme of the arch completed in one attempt are credible. In contrast, the biggest feature pertaining to the construction scheme completed in two attempts is that the constructed arch structure will produce uneven deformation in the original central partition of the wall area, and the settlement in this area will increase significantly after the central partition wall is removed. This is attributed primarily to the scheme of construction completed in two attempts. From one viewpoint, the deformation at the joints of the arches constructed in two attempts will be inconsistent, thus resulting in construction joints. From another viewpoint, the arch cannot completely transfer the load to the arch feet on both sides under the support of the central partition wall. Therefore, a larger deformation will inevitably appear at the supporting part after the two parts of the arch have been shut and the central partition wall has been removed.

From the above analysis, it can be inferred that the second lining of the arch completed in one attempt is better than that completed in two attempts in the case of the large-span PBA method. Although the effects of the two schemes on the ground deformation are almost similar, in terms of the stability of the arch structure, the scheme completed in two attempts is inferior to the scheme completed once. Therefore, in the construction of the large-span PBA method, the second lining of the arch construction should adopt the scheme of the arch completed in a single attempt.

## 5. Conclusions

Based on the large-span PBA method project of Qinghuadongluxikou Station of Beijing Metro Line 15, this paper systematically studies the response characteristics of surface deformation under different construction stages by numerical simulation combined with field measurement. Then the influence of three different excavation factors on surface deformation in the construction stage of the pilot tunnel is simulated and analyzed. Finally, two schemes of arch secondary lining structure are simulated and compared. The following are the primary conclusions:

1. During the excavation of the pilot tunnel of the station, the development of the settlement groove yields the obvious multicavern effect, and dual and single grooves appear alternately after different pilot tunnel excavations. Compared with the lower pilot tunnel excavation, the effects of upper pilot tunnel excavation on ground settlement and surface deformation are more apparent, and the development of settlement groove is more severe.
2. It is obvious that the development of ground settlement groove progresses in stages during the construction of the large-span PBA method. Through the numerical simulation, it is concluded that the settlement ratio of pilot tunnel excavation: pile-beam construction: arch initial support: arch secondary lining is about 5:1.1:3.3:0.6. Settlement deformation mainly occurs in pilot tunnel excavation stage. Therefore, the actual construction should focus on the prevention and control of ground subsidence during the excavation stage of the pilot tunnel.
3. The comparison of the field monitoring findings and the numerical simulation results reveal that the two are extremely compatible, confirming the accuracy of the numerical simulation results.
4. By comparing and analyzing six groups of pilot tunnel excavation schemes, the optimal pilot tunnel excavation scheme of large span PBA method is established, which excavates the middle first and then excavates both sides, and first through the upper layer and then through the lower layer.
5. The second lining of the arch completed in a single attempt was better than that completed in two attempts in the case of the large-span PBA method. Although the effects of the two schemes on the ground deformation are almost similar, in terms of the stability of the arch structure, the scheme completed in two attempts will lead to an uneven distribution of stress. In addition, greater ground deformation is caused by the removal of the central partition wall and will result in the construction of arch joints in the case in which construction was completed in two attempts.

This study accurately simulates the ground deformation law caused by the construction of large-span PBA method. Therefore, the numerical model proposed in this paper can be used in similar engineering applications. The research results enrich the knowledge of the influence of large-span PBA construction on the surface deformation of subway stations in silty clay–pebble composite strata. The optimal pilot tunnel construction scheme and buckle arch construction scheme obtained in this paper provide a reference for the design and construction of similar PBA subway stations.

## Figures and Tables

**Table 1:** Soil properties.

Soil	Thickness (mm)	Density *ρ* (kg/m^3^)	Bulk Modulus *K* (MPa)	Shear Modulus *G* (MPa)	Cohesion *C* (kPa)	Internal Friction Angle *φ* (°)
Filling	2300	1750	10.3	3.9	10	10
Silty clay	6540	1970	14.5	6.3	19	17
Silty clay	7270	1960	25.0	10.2	22	16
Silty clay	7800	1980	31.6	13.7	26	18
Pebbles	6000	2020	27.8	18.3	10	40
Silt	8000	1980	25.0	11.5	25	22
Pebbles	11,800	2050	29.8	20.5	10	40
Pebbles	30,290	2080	30.0	20.6	10	45

**Table 2:** The terrain and climate characteristics of the area where the subway station is located.

Terrain	Impact of Unfavorable Geology	Climate	Climatic Characteristics	Weather Disaster
Plain card country	The partially saturated sand and silt layers at the top of the station are prone to quicksand and quicksand.	Temperate monsoon	Summer is hot and rainy; winter is cold and dry.	Precipitation, low temperature

**Table 3:** Physical and mechanical parameters of supporting structure.

Structural Material Type	Structure	Elastic Modulus E (MPa)	Poisson’s Ratio ν	Density (kg/m^3^)
Pregrouting	Grouting reinforcement ring	15,000	0.28	2300
C20 concrete	Concrete backfill	25,500	0.20	2400
C20 grid steel frame Reinforced concrete	Pilot tunnel support	26,000	0.20	2500
	Primary lining			
I-beam (equivalent to the wall)	Middle wall steel support	6000 (equivalent to the wall)	0.29	7900
C30 concrete	Crown beam	30,000	0.20	2500
	Strip foundation			
	Side pile			
C40 concrete	Top stringer	32,500	0.20	2500
	Bottom stringer			
	Secondary lining			
	Middle floor slab			
	Station floor slab			
Steel pipe pile and C50 concrete	Center column	59,000	0.20	3200

**Table 4:** Specific scheme of working conditions.

Plan Number	Detailed Construction Plan	Influencing Factor
Plan 1	(4, 6→5) after penetration→(1, 3→2) after penetration	Whether the pilot tunnel of each layer is through construction respectively: (1) plan 1 contrast plan 2; (2) plan 3 contrast plan 4 Horizontal excavation sequence: plan 2 contrast plan 5 contrast plan 6 Vertical excavation sequence: plan 2 contrast plan 4
Plan 2	4, 6→5→1, 3→2	
Plan 3	(1, 3→2) after penetration→(4, 6→5) after penetration	
Plan 4	1, 3→2→4, 6→5	
Plan 5	5→4, 6→2→1, 3	
Plan 6	4→6→5→1→3→2	

**Table 5:** Summary of fitting results.

Plan Number	Maximum Surface Settlement *S_max_* (mm)	Backbend Distance *i* (m)	Fitting Correlation Coefficient R^2^	Formation Loss Rate V_1_ (%)
Plan 1	−37.221	12.655	0.9802	0.00906
Plan 2	−38.735	12.926	0.9840	0.00963
Plan 3	−34.536	12.304	0.9764	0.00817
Plan 4	−36.021	12.524	0.9769	0.00869
Plan 5	−37.962	12.905	0.9796	0.00942
Plan 6	−39.979	13.125	0.9838	0.01009

## Data Availability

The data presented in this study are available upon request from the corresponding author.

## References

[1] Zhang J.H., Xu X.M., Hong L., Wang S.L., Fei Q. (2011). Networked analysis of the Shanghai subway network, in China. Phys. A Stat. Mech. Its Appl..

[2] Peng Y.-T., Li Z.-C., Schonfeld P. (2019). Development of rail transit network over multiple time periods. Transp. Res. Part A Policy Pract..

[3] Choi J.-Y., Kim S.-H., Lee H.-H., Chung J.-S. (2021). Improvement of automatic measurement evaluation system for subway structures by adjacent excavation. Materials.

[4] Kim D., Pham K., Park S., Oh J.Y., Choi H. (2020). Determination of effective parameters on surface settlement during shield TBM. Geomech. Eng..

[5] Liu T., Wang H.T., Su X.T., Li K.X., Liu S.Q., Guo F.S. (2020). Instability mechanism of cavity-bearing formation under tunnel excavation disturbance. Adv. Civ. Eng..

[6] Ding Z., Wei X.-J., Wei G. (2017). Prediction methods on tunnel-excavation induced surface settlement around adjacent building. Geomech. Eng..

[7] Xiang Y.Y., He S.H., Cui Z.J., Ma S.Z. (2005). A subsurface “drift and pile” protection scheme for the construction of a shallow metro tunnel. Tunn. Undergr. Space Technol..

[8] Wu D., Deng T.F., Zhao R.K., Wang Y.H. (2018). THM modeling of ground subsidence induced by excavation of subway tunnel. Comput. Geotech..

[9] Sharifzadeh M., Kolivand F., Ghorbani M., Yasrobi S. (2013). Design of sequential excavation method for large span urban tunnels in soft ground—Niayesh tunnel. Tunn. Undergr. Space Technol..

[10] Sharifi A., Hosseingholizadeh M. (2019). The effect of rapid population growth on urban expansion and destruction of green space in Tehran from 1972 to 2017. J. Indian Soc. Remote Sens..

[11] Sharifi A., Mahdipour H., Moradi E., Tariq A. (2022). Agricultural field extraction with deep learning algorithm and satellite imagery. J. Indian Soc. Remote Sens..

[12] Zhang G.H., Chen H.Y., Deng K., Tong J.J., Ma X.Y. (2019). Comparison of Chongqing metro Station construction method in super-large section tunnel of the stratigraphic strata. Urban Mass Teansit.

[13] Yu L., Zhang D.L., Fang Q., Cao L.Q., Xu T., Li Q.Q. (2019). Surface settlement of subway station construction using Pile-Beam-Arch approach. Tunn. Undergr. Space Technol..

[14] Xu Y.S., Tang B., Duan Y.W. (2020). Research on surface settlement of subway station construction using Pile-Beam-Arch approach. IOP Conf. Series Earth Environ. Sci..

[15] Li T., Zhang Z.Y., Luo M.C., Liu B., Wang Y.L., Li L.F. (2022). Analytical solution of loosening pressure model for shallow tunnel based on pile-beam-arch method. KSCE J. Civ. Eng..

[16] Liu W., Luo F., Mei J. (2000). A new construction method for a metro station in Beijing. Tunn. Undergr. Space Technol..

[17] Huang B., Du Y.H., Zeng Y., Cao B., Zou Y., Yu Q. (2022). Study on stress field distribution during the construction of a group of tunnels using the pile-beam-arch method. Buildings.

[18] Li T., Wang L.Y., Wang Y., Liu S., Li Y.Y., Li B.R. (2017). Influence of step lengths of pilot tunnel excavation on surface subsidence based on PBA method. J. Jinan Univ. Sci. Technol..

[19] Guan Y.-P., Zhao W., Li S.-G., Zhang G.-B. (2014). Key techniques and risk management for the application of the pile-beam-arch (PBA) excavation method: A case study of the Zhongjie subway station. Sci. World J..

[20] Liu X.R., Liu Y.Q., Qu W.B., Tu Y.L. (2016). Internal force calculation and supporting parameters sensitivity analysis of side piles in the subway station excavated by Pile-Beam-Arch method. Tunn. Undergr. Space Technol..

[21] Zhang M.J., Liu Y., Fan L.F., Li P.F. (2017). Performance of constructing a double-deck subway station by combining the shield method and cavern-pile method. Tunn. Undergr. Space Technol..

[22] Liu X.R., Liu Y.Q., Yang Z.P., He C.M. (2017). Numerical analysis on the mechanical performance of supporting structures and ground settlement characteristics in construction process of subway station built by Pile-Beam-Arch method. KSCE J. Civ. Eng..

[23] Guo X.Y., Wang Z.Z., Geng P., Chen C.J., Zhang J. (2021). Ground surface settlement response to subway station construction activities using Pile-Beam-Arch method. Tunn. Undergr. Space Technol..

[24] Liu J., Wang F., He S.H., Wang E., Zhou H. (2015). Enlarging a large-diameter shield tunnel using the Pile-Beam-Arch method to create a metro station. Tunn. Undergr. Space Technol..

[25] Jia P.-J., Zhao W., Chen Y., Li S.-G., Han J.-Y., Dong J.-C. (2018). A case study on the application of the steel tube slab structure in construction of a subway station. Appl. Sci..

[26] Su J., Fang Q., Zhang D.L., Niu X.K., Liu X., Jie Y.M. (2018). Bridge responses induced by adjacent subway station construction using shallow tunneling method. Adv. Civ. Eng..

[27] Jiang B.F., Jia P.J., Zhao W., Wang W.T. (2018). The application of compressive sampling in rapid ultrasonic computerized tomography (UCT) technique of steel tube slab (STS). PLoS ONE.

[28] Pan X.M., Lei C.H. (2012). Comprehensive Engineering Technology for Sand and Pebble Conglomerate Strata of Beijing Metro.

